# Nutritional Knowledge, Attitudes, and Practices among Family Physician Practitioners in Gulf Countries (Bahrain, Kuwait, Saudi Arabia, and UAE)

**DOI:** 10.3390/healthcare11192633

**Published:** 2023-09-27

**Authors:** Abeer S. Alzaben, Abeer A. Aljahdali, Lulua F. Alasousi, Ghadeer Alzaben, Lynne Kennedy, Anwar Alhashem

**Affiliations:** 1 Department of Health Sciences, College of Health and Rehabilitation Sciences, Princess Nourah bint Adulrahman University, P.O. Box 84428, Riyadh 11671, Saudi Arabia; asalzaben@pnu.edu.sa; 2 Department of Clinical Nutrition, Faculty of Applied Medical Sciences, King Abdulaziz University, P.O. Box 80215, Jeddah 21589, Saudi Arabia; aaoaljahdali1@kau.edu.sa; 3 Department of Nutritional Sciences, University of Michigan, Ann Arbor, MI 48109, USA; 4 Kuwait Family Medicine Board, Faculty of Primary Health Care, Kuwait Institute for Medical Specializations, Ministry of Health, Safat P.O. Box 1793, Kuwait; lfalasousi@moh.gov.kw; 5 Department of Surgery, Awali Hospital, Awali P.O. Box 2555, Bahrain; ghadeer_alzaben@bapco.net; 6 Public Health and Nutrition, College of Natural and Health Sciences, Zayed University, Abu Dhabi P.O. Box 144534, United Arab Emirates; lynne.kennedy@zu.ac.ae

**Keywords:** nutrition, knowledge, attitude, practice, family physician, GCC

## Abstract

Family physicians serve as pivotal points of contact within global healthcare systems. Nutrition plays a significant role in lifestyle and preventive medicine. With the rise of chronic and non-communicable diseases in Gulf Cooperation Council (GCC) countries, these physicians advise patients and the public on diet-related conditions. This descriptive cross-sectional study assessed the nutritional knowledge and practices across the GCC. Combining survey data from multiple GCC sites, a sample of family physicians was generated. Registered GCC family physicians received email invitations containing participant information, informed consent forms, and a self-administered online questionnaire. Analysis of data from four GCC countries involving 8751 family physicians and nutrition experts revealed an average nutrition knowledge score of 62%. Nearly all participants recognized the importance of nutrition in preventing and treating chronic diseases. Common nutritional practices included recommending regular exercise (92%), referring patients to dietitians for nutrition-related concerns (70%), and providing nutrition advice (68.6%). GCC family physicians underscore the significance of nutrition in preventing and managing chronic ailments. Therefore, incorporating nutritional counseling into their practices is essential. This study highlights the need to integrate nutrition education into medical curricula and ongoing professional development, given that only 62% of GCC family physicians correctly answered nutrition-related questions.

## 1. Introduction

Family physicians are globally recognized as the primary point of contact and gatekeepers to the healthcare system, excluding emergency care. They play a crucial role in providing preventative medicine and facilitating access to planned medical care for individuals in need [1]. Healthcare includes planned treatment, medical therapy, and maintaining a healthy lifestyle to prevent various non-communicable diseases (NCDs) [1]. Nutrition constitutes a pivotal aspect of lifestyle and preventative medicine. Inadequate dietary intake stands out as one of the most prevalent risk factors affecting long-term health. It is closely associated with primary causes of preventable conditions and NCDs, including obesity (itself both a comorbidity and an important risk factor), type 2 diabetes, and cardiovascular disease [2]. As the Gulf Cooperation Council (GCC) countries display signs of a similar epidemiological transition to other developed high-income nations, the burden of chronic or NCDs continues to rise at an alarming rate. The demand for healthcare services and their associated costs have also surged. Consequently, there is an urgent need for more preventive policies aimed at addressing nutritional and diet-related risk factors. Given that many of these NCDs are linked to dietary factors, physicians must be proficient in advising the public and their patients on risk prevention and chronic disease management through dietary adjustments.

Due to the substantial consequences associated with malnutrition or unhealthy dietary patterns, it is critical that family physicians possess both competence and confidence in discussing the impact of dietary intake on patients’ health and well-being. Equally important is their ability to provide scientifically up-to-date evidence-based recommendations to patients at the earliest stage [3]. Serving as gatekeepers to primary and secondary health services, family physicians have the advantage of regularly interacting with numerous patients and their families. This unique position allows them to monitor health and lifestyle changes over time and identify signs of malnutrition or potential diet-related health issues that require attention. Therefore, family physicians must acquire and continually update the appropriate knowledge during their medical training and continuous practice to deliver clear and accurate nutritional recommendations to their patients [3]. This mandate is clearly articulated, as seen in the Third Heelsum International Workshop and various national-level professional bodies’ and medical councils’ guidelines for medical doctors and family physicians’ training [4,5]. Despite this high-level directive, numerous published reports indicate that medical students’ grasp of nutrition varies at best, and in cases such as the USA, Canada, and Saudi Arabia, it might be severely lacking [5,6,7,8]. This undermines the important role of family physicians in primary prevention, which involves addressing nutrition- and diet-related risk factors for NCDs in primary care settings. Furthermore, studies on licensed physicians (medical graduates) conducted in Saudi Arabia, Kuwait, and the USA [6,7,8,9] indicate a deficiency in their nutritional knowledge. Additional research on physicians in Arab countries, including Kuwait, Saudi Arabia, Qatar, and Azerbaijan [9,10,11,12], reports that over half (50–60%) of family physicians possess inadequate or poor levels of nutritional knowledge. In addition, studies exploring perceptions and attitudes regarding nutrition and nutrition counseling strategies among family physicians [11,13,14,15,16] are insufficient in terms of quantity and diversity. An assumption prevalent in the literature suggests that a ‘good’ knowledge of nutrition is likely to translate into positive attitudes toward physicians’ roles in nutrition-related primary prevention. Nonetheless, a significant critique of the literature on medical students’ or physicians’ nutritional knowledge is the lack of consensus on expected competencies or standards, as well as the absence of universal instruments for effectively assessing or rating their nutrition-related knowledge.

The GCC comprises six member states in the Arab region: Bahrain, Kuwait, Oman, Qatar, Saudi Arabia, and the UAE. The present study includes the first four of these states. Whereas all Arab GCC member states share social, political, and cultural similarities, they are governed independently [17]. Education, state registration, licensing, and physician regulation, although similar in each country, are also governed independently. However, the medical education curricula are comparable across the GCC [18]. Despite some studies conducted previously, which date back many years, there is a scarcity of current information regarding nutritional knowledge among physicians, especially family physicians, in this region. Additionally, previous studies assessing the nutritional knowledge, attitudes, and counseling practices of family physicians in this area have faced criticism due to poor response rates, restricting the generalizability of their findings. This study aims to address these gaps by collecting and consolidating data from family physicians in several GCC countries. Through this approach, it seeks to provide a more comprehensive and up-to-date assessment of the prevailing situation. This study endeavors to evaluate the nutritional knowledge, attitudes, and practices of family physicians in selected Gulf countries (Bahrain, Kuwait, Saudi Arabia, and the UAE). By doing so, this study provides current insights into the nutritional capability of existing family physicians in the Gulf region. It identifies areas that may benefit from curricular enhancements, with the overarching goals of preventing diet-related chronic diseases and promoting the nutritional well-being of the population.

## 2. Materials and Methods

A descriptive cross-sectional study was conducted to assess the nutrition knowledge and practices of practicing family physicians in GCC countries. The multicenter cross-GCC research collaboration aimed to aggregate survey data to form a representative sample of family physicians across the GCC region. A detailed study protocol was provided to the collaborating centers to ensure consistency and transparency across research sites.

### 2.1. Participants and Recruitment

All registered family physicians employed in any GCC member state at the time of this study were eligible to participate. Physicians who were retired, unemployed, or unregistered during the survey period were excluded. Due to ethical and practical constraints, direct contact with family physicians was not possible. Instead, gatekeepers were relied upon to disseminate study details and the survey. We contacted all active family physicians registered with their respective licensing or governing bodies. In Saudi Arabia, physicians are registered with the Saudi Commission for Health Specialties; in Kuwait, with the Ministry of Health; in Bahrain, with the National Health Regulatory Authority of Bahrain; and in the UAE, with the Dubai Health Authority and Abu Dhabi Health Authority, the latter also supporting Sharjah.

### 2.2. Data Collection

Data collection occurred over one year (2021–2022). All family physicians registered with the respective agencies in the GCC received an email containing comprehensive study information, informed consent, and a self-administered online questionnaire.

### 2.3. Research Instrument

The research instrument, developed before 2005 [10] and used in Middle Eastern studies, underwent reliability testing.

The survey consisted of four sections: (i) sociodemographic characteristics, including age, nationality, sex, years since graduation, professional experience, workplace, and country of medical school; (ii) self-reported nutrition knowledge and information sources; and a nutrition knowledge section with 16 multiple-choice questions evaluating nutritional knowledge on topics such as general nutrition, nutrition related to chronic diseases, and specialized nutrition. Each question had three answer choices, and correct responses were scored. The highest possible knowledge score was 16; (iii) the Nutritional Attitudes section used a five-point Likert scale to assess opinions about nutrition in primary care; and (iv) the Nutritional Practice section included seven statements evaluating nutritional practices, with five possible response options: all patients, the majority of patients, some patients, or a minority of patients, and never. See Appendix A.

### 2.4. Reliability and Validity

The research instrument, originally developed before 2000 and previously used in Middle Eastern studies, underwent reliability testing. Cronbach’s alpha indicated acceptable internal consistency for family physicians’ nutritional practice (0.74 for 7 questions), attitudes (0.70 for 4 questions), and the 16 questions in the knowledge section (0.50).

### 2.5. Statistical Analysis

Descriptive analyses presented means (standard deviations) for continuous variables and frequencies (percentages) for categorical variables. The Shapiro-Wilk test was employed to examine the normal distribution assumption for continuous variables, with a *p*-value < 0.05 indicating a violation. Wilcoxon rank-sum and Kruskal-Wallis tests were used for non-normally distributed continuous variables with binary and ≥3-level categorical variables, respectively. Statistical tests were two-sided at a significance level of 0.05. When the overall *p*-value from the Kruskal-Wallis test indicated significance (*p* < 0.05), a post-hoc test was performed to determine which specific groups differed from each other. Spearman’s correlation was used to assess associations between two continuous variables when at least one variable had a non-normal distribution. All statistical analyses were performed using Statistical Analysis Software (SAS) (SAS Institute, Cary, NC, USA), version 9.4 [19]. For all tests, statistical significance was established at a *p*-value of less than 0.05.

### 2.6. Ethical Considerations

Institutional Review Board (IRB) approvals were obtained from the governing bodies of each academic institution in the respective GCC countries: Bahrain (Approval with No. IRB#), Kuwait (IRB# 2021/1891), Saudi Arabia (IRB# 21-0170), and the UAE (IRB# ZU21_126_F). Separate IRB approval was required to survey physicians in the UAE, which was obtained from the relevant professional institutions responsible for maintaining the register of family physicians in each of the UAEs regions, including Dubai, Abu Dhabi, and Sharjah.

Ethical procedures, encompassing data storage and governance, were adopted. Although the risk to human participants is minimal or nonexistent, we recognize that confidentiality and anonymity hold paramount importance for professionals. To address this concern, potential participants were assured via email and participant information that the utilization of the Google form ensured the collection of data without personal identification, thereby guaranteeing anonymity.

## 3. Results

Data were collected and aggregated from four of the six GCC-member states: Bahrain, Kuwait, Saudi Arabia, and the UAE. Response rates for each country were as follows: 6014 responses from Saudi Arabia (2.6% of all registered family physicians), 1200 responses from Kuwait (5.4% of registered physicians), 278 responses from Bahrain (6.8% of registered physicians), and 1259 responses from the UAE (5.4% of registered physicians). This aggregation resulted in a total sample of N = 8751 practitioners across the four GCC member states.

Table 1 presents the aggregated data of respondents who participated in this study. Within the aggregated study sample, Saudi Arabia had the highest representation at 58.3%, with more than 1 in 2 family physicians responding. Kuwait had a response rate of 1 in 4, while in the UAE and Bahrain, 1 in 10 or fewer family physicians participated. Of the respondents, the majority (59%) were female, and over half (57%) had received their medical degree from Gulf countries. The average age was 40.33 ± 9.84 years, with an average of 14.33 ± 9.93 years since graduation from medical school. A quarter of respondents reported 5–9 years of professional experience post-graduation, and approximately 52% reported working in primary healthcare facilities (Table 1).

Table 2 provides insight into self-reported nutrition-related knowledge and sources. When asked if they possessed knowledge of nutrition, the majority (80%) of the physicians responded positively, attributing their knowledge primarily to the medical curriculum (35.98%) and non-curriculum activities such as seminars, lectures, short courses, and conferences (30.68%).

The nutrition-related knowledge scores are presented in Table 3 and were calculated by cumulatively adding the correct responses to the 16 knowledge questions. The mean score was 10.63 ± 2.35, ranging from 2 to 15 out of a possible score range of 0 to 16 points. Notably correct responses were observed for questions such as the association of folate with neural tube defect prevention (95%), the preventive role of fruits and vegetables against cancer (91%), the protective role of potassium against hypertension (86%), and the recommended calcium intake of 1200 milligrams/day for adults aged 51–70 years (80%).

However, there were notable incorrect answers to questions regarding the effect of excess protein intake on calcium loss (68%), the role of zinc as an antioxidant nutrient (48%), the major type of fat in olive oil being monounsaturated fat (46%), and the effectiveness of short-term diet plans in causing weight loss due to the body losing water (45%) (Table 3).

Table 4 shows the response frequencies to the four questions used to assess nutrition-related attitudes. The majority of respondents strongly agreed with the four nutrition-related statements (*n* = 78–95%). The statement with the highest agreement was “Nutrition is a significant component in the prevention and progression of many chronic diseases” (95%). While only 9% of participants disagreed with the negative (reverse) statement, “I feel that patients want more information on nutrition than I can provide”.

Table 5 outlines how respondents’ practices aligned with seven areas of nutrition-related practices, ranging from 27% to 92%. The most frequently reported nutrition practices, delivered “for most patients” by respondents, were (i) advising taking regular exercise (92%), (ii) offering nutrition advice (69%), and (iii) assessing patients’ height and weight to calculate body mass index (67%). Additionally, 70% reported referring patients to a dietitian for nutrition-related disorders, while 66% reported “asking most of their patients about their dietary intake as a routine preventive strategy.” The least utilized practices included educating patients on nutrition fact labels (27%) and requesting patients to maintain food diaries (31%).

The bivariate associations between the overall nutrition knowledge score and sociodemographic variables are presented in Table 6. Those working in private hospitals had the highest overall nutrition knowledge score (11.95 ± 1.99), compared to those in governmental or Ministry of Health hospitals (10.13 ± 2.46) or primary health care (10.80 ± 2.22) (*p*-value = 0.0070). The analysis suggested that working in private hospitals was significantly associated with a higher nutrition knowledge score than in governmental or Ministry of Health hospitals (*p*-value = 0.0101). Moreover, the origin of a medical degree was positively correlated with overall nutrition knowledge scores. Graduates from international countries displayed a mean overall score of 11.45 ± 2.03, while general practitioners trained in Gulf countries and other Arab countries showed scores of 10.57 ± 2.29 and 10.12 ± 2.53, respectively (*p*-value = 0.0171). Post-hoc tests showed that degrees from international countries were significantly associated with a higher nutrition knowledge score than counterparts with degrees from other Arab countries (*p*-value = 0.0127). A positive relationship was observed between self-reported nutrition knowledge and overall nutrition knowledge scores. The mean overall score of nutrition knowledge was 10.85 ± 2.33 for those reporting nutrition knowledge and 9.74 ± 2.21 for those without it (*p*-value = 0.0009) (Table 6).

## 4. Discussion

The objectives of the present study were to assess current levels of nutritional knowledge, attitudes, and nutrition-related practices among family physicians in the GCC region. Among the six GCC member states, Bahrain, Kuwait, Saudi Arabia, and the UAE met recruitment criteria (N = >3% registered family physicians) for inclusion in the final analysis. A notable strength lies in the established research collaboration across GCC member states, leveraging shared socio-cultural traits and consistent socio-political and cultural contexts, resulting in aggregated data from each state and improving response rates in a challenging study demographic. To our knowledge, the present study is the first to simultaneously assess nutritional knowledge among family physicians in several GCC countries, particularly pertinent due to the uniform medical curriculum across GCC medical schools [18]. Another strength lies in the uniform research protocol and questionnaire across research sites and countries.

Our findings suggest that, during this study period, approximately two-thirds of employed family physicians in the GCC could be considered knowledgeable in nutrition as it relates to the primary care setting. The average overall nutrition knowledge score of family physicians was 62%. Higher nutrition knowledge scores were observed among family physicians working in private hospitals and among those who graduated from universities outside the GCC (i.e., international institutions). In private hospitals, family physicians may have more time to engage with their patients and provide nutritional counseling. This may provide them with more opportunities to learn about the dietary needs of their patients and develop nutrition expertise. In addition, international medical school graduates may be required to complete a nutrition course that would provide them with a solid foundation in the principles of nutrition counseling. Physicians who self-reported good nutrition knowledge also demonstrated higher levels of nutrition knowledge in the survey. In our survey, nearly all GCC physicians (95%) were aware of the link between inadequate folate intake and the prevention (risk reduction) of Neural Tube defects, the preventative role of fruits and vegetables, and various types of cancer (91%). Moreover, 86% recognized the potential protective role of potassium against hypertension, and 80% acknowledged the beneficial effects of adequate calcium intake for older individuals. These concepts are highly pertinent and suitable for primary prevention efforts, aligning with the findings of previous studies. However, a notable proportion (68%) inaccurately recognized the relationship between excess protein intake, calcium loss, and osteoporosis. Additionally, nearly half (48%) incorrectly identified zinc as an antioxidant nutrient. Similarly, 46% erroneously associated monounsaturated fat as a major component of olive oil, suggesting that global evidence advocating for greater emphasis on fat types rather than quantity is not effectively reaching frontline health workers such as physicians. This has significant implications for preventative medicine. Additionally, 45% of respondents incorrectly concurred with the statement that short-term (diet) plans are successful methods for achieving weight loss because they lead to water loss in the body. This underscores potential misinformation or a lack of awareness regarding weight management principles and options among many GCC physicians. This is particularly concerning considering the current and projected trends in the prevalence of nutrition-related NCDs, cardiovascular disease, diabetes, and related conditions or risk factors, including obesity, in the GCC population. Therefore, we urge those involved in medical education to address potential areas of misinformation and lack of awareness to safeguard the population’s health.

The current study demonstrates an average nutritional knowledge score of 62%. Previous studies conducted in Riyadh and Jeddah in 2004 and 2009 reported a nutritional knowledge level of approximately 52% [3,7]. Another recent study among intern physicians in Jeddah found an average of 55.6% correct answers to nutrition knowledge questions [20]. In Kuwait in 2013, using the same nutritional knowledge questionnaire as the current study, the average nutritional knowledge was found to be 60% [9]. A cross-sectional study in Iran revealed that less than 10% of medical students possessed adequate nutrition knowledge [11]. A recent systematic narrative review reported that nutrition knowledge scores among physicians and nurses typically ranged from 33% to 72% [21]. In addition, the majority of the patients in our study were from Saudi Arabia. The results of the current study surpass those of previous research conducted in Saudi Arabia in 2004 and 2009 while falling within the range of scores reported in the review.

This study further identified that family physicians working in private hospitals and those who graduated from international institutions exhibited higher nutritional knowledge. Comparable findings have been cited; for example, a systematic narrative review highlighted associations between greater nutrition knowledge and factors such as immigration, years of professional experience, and additional nutrition education and/or training [21]. In light of these findings, medical education authorities in the GCC might consider exploring nutrition curricula from other countries.

Globally, the significance of family physicians in NCD prevention through nutrition is well established. Consequently, it is unsurprising that, despite the aforementioned misinformation, a substantial majority (95.4%) of respondents in our study acknowledged the importance of nutrition in preventing and treating chronic diseases. This finding aligns with similar studies, such as [22,23]. Nevertheless, only 9% of respondents disagreed with the negative statement *“I feel that patients want more information on nutrition than I can provide”* (Q.3, Table 4), indicating that a majority (>90%) of GCC physicians may lack the confidence to deliver nutrition information effectively in the primary care setting. This resonates with previous studies wherein family physicians recognize the importance of nutrition advice and counseling in primary care but express inadequate confidence or self-efficacy in their delivery, regardless of their nutrition knowledge. Typical barriers to providing nutrition counseling by family physicians include limited time, patient non-adherence to dietary recommendations, and gaps in knowledge and resources [23,24]. In the domain of preventative medicine, effective and confident communication or role modeling is critical for successful counseling or behavioral modifications. The lack of confidence among numerous frontline physicians in delivering nutrition counseling could potentially undermine ongoing initiatives aimed at addressing critical public health issues, such as obesity, through dietary modification and weight management. To ensure that graduates feel empowered to implement nutrition-related practices as part of preventive medicine, the medical curriculum should encompass comprehensive and up-to-date nutrition content.

When asked to rank the frequency of engaging in seven areas of nutrition practice commonly expected of family physicians’, the activity most frequently undertaken by respondents in the present study was advising patients to exercise regularly (92%). This was closely followed by ‘Referring patients to a dietitian if they have a nutrition-related disorder’ (70%) and ‘providing nutrition advice’ (68.6%), demonstrating a promising inclination among GCC physicians to offer nutritional guidance as frequently as they refer patients to specialists (dietitians), suggesting an acknowledgment of their scope of practice rather than avoidance. Slightly less frequent (66%) was the willingness of physicians to assess body mass index (BMI). Although inquiring about nutrition was fairly common, there was a clear reluctance and fewer occasions (>64%, sometimes) where physicians asked patients to maintain Food Diary. Additionally, most (1:2 consultations) physicians did not routinely advise patients on interpreting Nutrition (food) labels (47.7%,‘minority’). Given the importance of assessing the current diets as part of goal-setting and behavior modification strategies recommended for primary care professionals in diet-related prevention policies, this finding should be taken into consideration by those responsible for designing nutrition education and training for medical professionals. Possessing the skills and confidence to provide practical strategies for modifying dietary behavior, including enhancing nutritional literacy, is a key requirement for primary care professionals. Nutritional practices among family physicians vary in the literature [22,24]. These practices encompass measuring anthropometrics, calculating BMI, and providing nutritional advice [23]. A majority of studies have reported that family physicians typically refer patients to registered dieticians for relatively specific reasons, such as clinical nutrition counseling [15,16,22,23,24]. Studies conducted in Taiwan and Hawaii found that approximately 66–70% of primary care physicians reported utilizing nutritional or health modification strategies [15,16], yet many also expressed a need for more experience in employing basic nutritional practices and strategies [15].

It is firmly established that nutrition education is essential for primary health care workers, including family physicians, and thus should be incorporated into all medical curricula [25]. A recent study on medical interns in Saudi Arabia reported that half (50%) were satisfied with the nutritional content of their medical nutrition curriculum [20]. Additionally, physicians who believed they had good nutritional knowledge achieved higher nutritional knowledge scores. Nutrition therapy and management complement medical care in primary settings to prevent and treat chronic conditions such as hypertension and type 2 diabetes [25]. Limited nutritional knowledge among family physicians can have a negative impact on patient outcomes. For instance, patients with diabetes often seek advice from their family physicians on how to manage their blood glucose levels. However, if the physician lacks nutrition expertise, they may have difficulty providing accurate guidance, which can lead to unhealthy eating habits in patients, thereby worsening their diabetes condition. In the case of elderly patients, physicians may face difficulty diagnosing malnourishment due to a lack of nutritional knowledge. This can result in significant health problems for the patient, such as delayed wound healing and weakened muscular strength. Therefore, continuing medical education programs focused on nutrition are essential for physicians, emphasizing the role of healthcare providers in lifestyle modifications for preventing and treating chronic diseases [26].

This collaborative cross-country study was initiated in 2020, and following extensive preparation for multiple ethical approvals, data collection occurred between 2021 and 2022. The data in this study was collected through email surveys only. This approach may have resulted in low response rates in certain countries since some participants may have missed the email. It is possible that the email was filtered into their spam folders. Additionally, we acknowledge that the low response rates from some countries may have led to selection bias, which could impact the generalizability of this study’s findings.

## 5. Conclusions

In summary, based on the findings of the present survey, most family physicians in the GCC have positive attitudes toward the importance of nutrition in the prevention and treatment of chronic diseases in primary care. In addition, participants in the current study perceived nutritional counseling in the primary care setting as a fundamental responsibility of physicians. While a majority of family physicians in Saudi Arabia, Kuwait, the UAE, and Bahrain self-reported having nutrition knowledge, the survey results revealed that only two-thirds (62%) of GCC family physicians could correctly answer nutrition-related questions. In practice, most family physicians refer patients to dietitians for specific clinical nutrition-related disorders. Nutrition knowledge is associated with graduating from international institutions and working in private hospitals. This demonstrates the importance of nutrition education in medical schools and continuing medical education.

As for future direction, it is recommended that medical schools and residency programs dedicate more time to nutrition education in their curricula. Provide students and residents with opportunities to learn about nutrition from clinical dietitians and other nutrition experts. Furthermore, while developing CME activities for family physicians, it is recommended to focus on offering CME courses on nutrition that are relevant to the needs of family physicians and make CME courses on nutrition more accessible and affordable. Lastly, developing GCC clinical practice guidelines for nutrition that are based on the latest research and evidence, involving family physicians and clinical dietitians in the development of those guidelines, and providing courses on how to put them into daily clinical practice would lead to better nutritional counseling for patients and, ultimately, improved patient health outcomes.

## Figures and Tables

**Table 1:** Respondents’ characteristics (N = 264).

Sociodemographic Characteristics	N (%) Mean ± Standard Deviation
Study site	
Bahrain	19 (7.20)
Kuwait	66 (25.00)
Saudi Arabia	154 (58.33)
United Arab of Emirates (UAE)	25 (9.47)
Age (years)	40.33 ± 9.84
Sex	
Female	156 (59.09)
Male	108 (40.91)
Years since graduation	14.33 ± 9.93
Professional years of experience	
≤4	39 (14.77)
5–9	66 (25.00)
10–14	57 (21.59)
15–19	39 (14.77)
≥20	63 (23.86)
Current work sites (by type)	
Governmental or Ministry of Health hospitals	104 (39.39)
Private Hospitals	21 (7.95)
Primary Healthcare	139 (52.65)
Country of medical degree ^1^	
Gulf countries (Bahrain, Kuwait, Oman, Saudi Arabia, UAE)	150 (56.82)
Other Arab countries (Egypt, Jordan, Lebanon, Sudan, Syria, Yemen, and additional Arabic countries)	66 (25.00)
Other international countries: Bangladesh, Cuba, Europe, India, Ireland, Malta, Nigeria, Pakistan, Russia, Slovakia, UK, USA, Canada, and Asia (India, Pakistan, Iran, China)	47 (17.80)

^1^ N = 263 (missing 1 observation).

**Table 2:** Respondents’ self-reported nutrition-related knowledge (N = 264).

	N (%)
**Self-reported nutritional knowledge**	
Yes, I know about nutrition	210 (79.55)
No, I do not know about nutrition	54 (20.45)
**Sources of nutritional knowledge ^1^**	
Medical curriculum	95 (35.98)
Non-curriculum activities such as nutrition seminars, lectures, courses, and conferences	81 (30.68)
Journal articles and unspecified other sources of knowledge	63 (23.86)

^1^ N = 239 (missing 25 observations).

**Table 3:** Respondents’ knowledge of nutrition (N = 264).

Question	Correct	Incorrect
	N (%) (SD)	N (%) (SD)
1. Dietary fiber lowers blood cholesterol levels.	163 (61.74)	101 (38.26)
2. Excess of which nutrient may increase body calcium loss?	85 (32.20)	179 (67.80)
3. A nutrient believed to help prevent thrombosis is:	175 (66.29)	89 (33.71)
4. The adequate intake level of calcium for adults aged 51–70 years is:	212 (80.30)	52 (19.70)
5. The major type of fat in olive oil is:	143 (54.17)	121 (45.83)
6. Compared with unprocessed vegetable oil, hydrogenated fats contain:	173 (65.53)	91 (34.47)
7. Which nutrient is protective against hypertension?	226 (85.61)	38 (14.39)
8. Which vitamin is likely to become toxic if consumed in excessive amounts for an extended period of time?	152 (57.58)	112 (42.42)
9. The most concentrated source of vitamin B12 is:	181 (68.56)	83 (31.44)
10. Which substance raises the blood HDL-cholesterol level?	147 (55.68)	117 (44.32)
11. In general, dietary recommendations are intended to:	200 (75.76)	64 (24.24)
12. The type of food believed to have a preventive effect on various types of cancer is:	240 (90.91)	24 (9.09)
13. The number of kilocalories in one gram of fat is:	178 (67.42)	86 (32.58)
14. Which of the following is not an antioxidant nutrient?	136 (51.52)	128 (48.48)
15. The nutrient strongly associated with the prevention of neural tube defects is:	250 (94.70)	14 (5.30)
16. Short-term diet plans are usually successful in achieving weight loss because they:	144 (54.55)	120 (45.45)
Mean of the total knowledge score (Mean ± Standard deviation) (Min–Max)	10.63 ± 2.35 (2–15)	

**Table 4:** Respondents’ attitudes towards nutrition practice in primary care (N = 264).

Statement	(Strongly Agree + Agree)	Neutral	(Disagree + Strongly Disagree)
1. Nutrition is a significant component in the prevention and progression of many chronic diseases.	252 (95.45)	8 (3.03)	4 (1.52)
2. Nutrition counseling in the family practice setting is effective at changing patients’ behavior.	228 (86.36)	24 (9.09)	12 (4.55)
3. I feel that patients want more information on nutrition than I can provide.	205 (77.65)	35 (13.26)	24 (9.09)
4. Counseling patients about nutrition is one of the responsibilities of the physician.	224 (84.85)	31 (11.74)	9 (3.41)

Frequencies (%).

**Table 5:** Respondents’ ‘physician nutrition-related practice (N = 264).

Practice	All and Most Patients	Some Patients	Minority of Patients and Never
1. Assess the patient’s height and weight and calculate body mass index.	177 (67.05)	75 (28.41)	12 (4.55)
2. Ask patients about their dietary intake as a preventive strategy.	175 (66.29)	70 (26.52)	19 (7.20)
3. Offer nutritional advice.	181 (68.56)	73 (27.65)	10 (3.79)
4. Teach patients how to read a nutrition fact label.	72 (27.27)	66 (25.00)	126 (47.73)
5. Ask patients to maintain a food diary.	83 (31.44)	90 (34.09)	91 (34.47)
6. Refer patients to a dietician if they have a nutrition-related disorder.	185 (70.08)	63 (23.86)	16 (6.06)
7. Advise patients to exercise regularly.	243 (92.05)	18 (6.82)	3 (1.14)

Frequencies (%) are presented.

**Table 6:** Bivariate association between overall nutrition knowledge score and sociodemographic variables (N = 264).

	Correlation Coefficient Mean ± Standard Deviation	*p*-Value
Age (year) ^1^	0.04673	0.4496
Sex ^2^		0.3556
Female	10.70 ± 2.43	
Male	10.52 ± 2.22	
Length of time since graduation (year) ^1,3^	0.05569	0.3684
Professional years of experience ^4^		0.0986
≤4	10.00 ± 2.20	
5–9	11.14 ± 2.37	
10–14	10.60 ± 1.90	
15–19	10.38 ± 2.87	
≥20	10.65 ± 2.37	
Working sites ^4^		0.0070 *
Governmental or Ministry of Health hospitals **	10.13 ± 2.46	
Private hospitals **	11.95 ± 1.99	
Primary health care	10.80 ± 2.22	
Country of medical degree ^3,4^		0.0171 *
Gulf countries	10.57 ± 2.29	
Other Arab countries **	10.12 ± 2.53	
Other international countries **	11.45 ± 2.03	
Having nutritional knowledge ^2^		0.0009 *
No	9.74 ± 2.21	
Yes	10.85 ± 2.33	
Sources of nutritional knowledge ^4,5^		0.3036
Medical curriculum	10.93 ± 2.35	
Nutrition seminars, lectures, courses, and conferences	10.42 ± 2.37	
Journal articles and other sources	10.81 ± 2.44	

Means (standard deviations) are presented. ^1^ Spearman Correlation Coefficients. ^2^ *p*-value based on the Mann-Whitney U test. ^3^ N = 263 (missing 1 observation). ^4^ *p* -value based on the Kruskal-Wallis test. ^5^ N = 239 (missing 25 observations). * *p*-value < 0.05. ** Significant *p*-value (<0.05) for post-hock test for Kruskal-Wallis test.

## Data Availability

The data presented in this study are available on request from the corresponding author.

## Appendix A

The self-administered online questionnaire

**Questionnaire**
Section A: Socio-demographic Information

1- **Age: ………………………….**

2- **Nationality:**

- Saudi
- Qatari
- Kuwaiti
- Omani
- UAE
- Bahrain
- Other Nationality

3- **Gender**

a. male                                                                          b. female

4- **Length of time since graduation (year): ………………………….**

5- **Professional experience:**

- 0–4 years
- 5–9 years
- 10–14 years
- 15–19 years
- 20 years and above

6- **Work in:**

- governmental hospital (example: security forces hospital)
- ministry of health (example: king Saud medical city)
- private hospital (example: Dallah hospital)
- other (…primary health care……)

7- **Country of medical graduation?**

- Saudi Arabia
- Qatar
- Kuwait
- Oman
- UAE
- Bahrain
- other (……………………..)

8- **Do you have nutritional knowledge:**

a. Yes                                                                          b. No

9- **what is the sources of the nutritional knowledge**: (if your answer in question 7 was “yes” answer question 8) (3)

- Medical curriculum
- Nutrition seminars or lectures
- Nutrition courses designed for family physicians.
- Nutrition continuing education conferences.
- Journal articles
- Another source (……….)

Section B: Nutritional knowledge

1. **What type of dietary fiber is helpful in lowering the blood cholesterol level?**

- Soluble fiber.*
- Insoluble fiber.
- Cellulose.

2. **Excess of which nutrient may increase body calcium loss:**

- Protein.*
- Saturated fatty acid.
- Sugar.

3. **A nutrient believed to help prevent thrombosis is:**

- Omega-3 fatty acid.*
- Monounsaturated fat.
- Vitamin C.

4. **The adequate intake level of calcium for adult aged 51–70 years is:**

- 500 milligrams/day.
- 1200 milligrams/day.*
- 2000 milligrams/day.

5. **The major type of fat in olive oil is:**

- Saturated fat.
- Polyunsaturated fat.
- Monounsaturated fat.*

6. **Compared with unprocessed vegetable oil, hydrogenated fats contain:**

- More polyunsaturated fat.
- More trans fats.*
- More cholesterol.

7. **Which nutrient is protective against hypertension?**

- Potassium.*
- Chlorine.
- Iron.

8. **Which vitamin is likely to be toxic if consumed in excess amount for long period of time?**

- Vitamin C.
- Vitamin A.*
- Vitamin D.

9. **The most concentrated source of vitamin B12 is:**

- Fruit.
- Whole grain cereals.
- Meat.*

10. **Which substance raises the blood HDL-cholesterol level?**

- Animal protein.
- Riboflavin.
- Unsaturated fatty acid.*

11. **In general, dietary recommendations are intended to:**

- Maximize food efficiency.
- Maintain public health.*
- Increase athletic performance.

12. **Type of food believes to have a preventive effect on various types of cancer is:**

- Fruit and vegetable.*
- Milk.
- None of the above.

13. **The number of kilocalories in one gram of fat is:**

- 4
- 7
- 9*

14. **Which of the following is not an antioxidant nutrient?**

- Vitamin E.
- Beta-carotene.
- Zinc.*

15. **The nutrient strongly associated with the prevention of neural tube defects is:**

- Beta-carotene.
- Folate.*
- Vitamin C.

16. **Short-term (diet) plans are usually successful at achieving weight loss because they:**

- Decrease appetite.
- Cause the body to lose water.*
- Burn large amount of stored fat.

* the correct answer

Section C: Nutritional attitude (6).

To what extent you agree with the following statements, using a 5-point Likert scale (6).

Statement	Strongly agrees	Agree	Neutral	Disagree	Strongly disagrees.
Counseling patients about nutrition is one of the responsibilities of the physician.					
Nutrition is a significant component in the prevention and progression of many chronic diseases.					
Nutrition counseling in the family practice setting is effective at changing patients’ behavior.					
I feel that patients want more information on nutrition than I am able to provide.					

Section D: Nutritional practice (15).

How frequently do you practice the following on your patients?

Practice	All patients	Most patients	Some patients	Minority of patients	never
Assess the patient’s height and weight and calculate their BMI.					
Ask patients about dietary intake as a preventive strategy.					
Offer nutritional advice.					
Teach patients how to read a nutrition fact label.					
Ask patients to keep a food diary.					
Refer patients to a dietician if they have a nutrition-related disorder.					
Advise patients to exercise regularly.					
